# RETRACTED: Lee et al. Protective Effects of *Oenothera biennis* Against Hydrogen Peroxide-Induced Oxidative Stress and Cell Death in Skin Keratinocytes. *Life* 2020, *10*, 255

**DOI:** 10.3390/life16040656

**Published:** 2026-04-13

**Authors:** Seung Young Lee, Chul Hwan Kim, Buyng Su Hwang, Kyung-Min Choi, In-Jun Yang, Gi-Young Kim, Yung Hyun Choi, Cheol Park, Jin-Woo Jeong

**Affiliations:** 1 Nakdonggang National Institute of Biological Resources, 137, Donam 2-gil, Sangju-si 37242, Republic of Korea; nplsy001@nnibr.re.kr (S.Y.L.); kchul1204@nnibr.re.kr (C.H.K.); hwang1531@nnibr.re.kr (B.S.H.); kyungmc69@nnibr.re.kr (K.-M.C.); 2 Department of Physiology, College of Oriental Medicine, Dongguk University, Gyeongju 780-714, Republic of Korea; injuny@gmail.com; 3 Laboratory of Immunobiology, Department of Marine Life Sciences, Jeju National University, Jeju 63243, Republic of Korea; immunkim@jejunu.ac.kr; 4 Department of Biochemistry, Dong-Eui University College of Korean Medicine, Busan 47227, Republic of Korea; choiyh@deu.ac.kr; 5 Division of Basic Sciences, College of Liberal Studies, Dong-Eui University, Busan 47340, Republic of Korea

The journal retracts the article titled “Protective Effects of Oenothera biennis Against Hydrogen Peroxide-Induced Oxidative Stress and Cell Death in Skin Keratinocytes” [1], cited above.

Following publication, concerns were raised with the Editorial Office regarding the integrity of the data presented in this publication [1].

Adhering to our standard procedure, an investigation was conducted by the Editorial Office and Editorial Board that confirmed concerns related to the accuracy of the flow cytometric data presented in Figure 6. Although the authors fully cooperated with the investigation, the explanations and supporting materials provided were not deemed sufficient by the Editorial Board to resolve the identified concerns. Consequently, the Editorial Board has lost confidence in the reliability of the findings and decided to retract this publication [1], as per MDPI’s retraction policy.

This retraction was approved by the Editor-in-Chief of the *Life* journal.

The authors have been informed of this retraction but did not provide a comment on this decision.
